# A Modified Active Appearance Model Based on an Adaptive Artificial Bee Colony

**DOI:** 10.1155/2014/879031

**Published:** 2014-08-06

**Authors:** Mohammed Hasan Abdulameer, Siti Norul Huda Sheikh Abdullah, Zulaiha Ali Othman

**Affiliations:** ^1^Pattern Recognition Research Group, Centre for Artificial Intelligence Technology, Faculty of Information Science and Technology, Universiti Kebangsaan Malaysia, 43600 Bandar Baru Bangi, Malaysia; ^2^Department of Computer Science, Faculty of Education for Women, University of Kufa, Iraq; ^3^Data Mining and Optimization Group, Faculty of Information System and Technology, Universiti Kebangsaan Malaysia, 43600 Bandar Baru Bangi, Malaysia

## Abstract

Active appearance model (AAM) is one of the most popular model-based approaches that have been extensively used to extract features by highly accurate modeling of human faces under various physical and environmental circumstances. However, in such active appearance model, fitting the model with original image is a challenging task. State of the art shows that optimization method is applicable to resolve this problem. However, another common problem is applying optimization. Hence, in this paper we propose an AAM based face recognition technique, which is capable of resolving the fitting problem of AAM by introducing a new adaptive ABC algorithm. The adaptation increases the efficiency of fitting as against the conventional ABC algorithm. We have used three datasets: CASIA dataset, property 2.5D face dataset, and UBIRIS v1 images dataset in our experiments. The results have revealed that the proposed face recognition technique has performed effectively, in terms of accuracy of face recognition.

## 1. Introduction

Recently, biometric techniques have received a significant attention due to their wide applications in many areas such as security applications. Many examples of biometric techniques have been used such as face, voice, iris, hand geometry, retina, DNA, and ear [1–4]. Face recognition is a crucial biometric technique, which could be employed in several areas such as identification at front door for home protection and recognition at ATM or in combination with a smart card for authentication and video inspection for security [5–7]. Of late, the massive biometric applications have made face recognition as one of the dynamic research areas for computer and machine vision researchers [8]. It is noteworthy that the process of face recognition is difficult and challenging task, because it demands to consider all possible variations in the appearances, caused by change in illumination, facial characteristics, occlusions, and so forth. The major challenges in automatic facial recognition are face localization, feature extraction, and modeling [9–11].

Active appearance model (AAM) is one of the most prominent techniques [12] that has been widely used in feature extraction for many applications [13], which includes face modeling, studying human behavior, medical imaging tasks, like segmentation of cardiac MRIs or the diaphragm in CT data, and registration in functional heart imaging [14]. The active appearance model (AAM) details the appearance of the face [15–18] and it builds statistical model of shape and appearance of any given object. The model blends the constraints on both shape and texture by learning statistical generative models for the shape of a face and the appearance of a face. After creating the model, it is essential to fit the generated model to new images, which is important to find the accurate parameters of the model for an object [19]. Nevertheless, the deficiency of fitting result is the biggest concern while matching the model with original image [20]; moreover, selecting a fitting algorithm is a crucial issue of AAM [21]. The approaches employed for enhancing the fitting performance of AAM have been classified as four groups.

The first approach involves the usage of different versions of deformable model. Christoudias et al. [22, 23] have stated that the impact of light field manipulates human face from real images; therefore each prototype has been obtained by a specific view of 2D shape of each face under a specific light field. In terms of fitting the light-field model to the input image, they have first chosen a particular view of the light-field model, which is the closest to the view of the input image, and then used the direct search to match the input image to a point in the light-field manifold. Based on the application of manifold estimation in the unified view-based feature spaces [24] have proposed a technique for synthesizing face across poses, which is capable of synthesizing unseen views, even for huge variations in the poses. However, they have just tested their work on twenty-five 3D models, which is not adequate to validate the proposed approach.

The second approach for enhancing the fitting performance of AAM combines the existing models. In this context, [25] hybridized active shape models (ASMs) and active appearance models (AAMs), for reliable image interpretation. Moreover, [26] proposed texture-constrained active shape model (TC-ASM), which stemmed from the local appearance model of active shape model (ASM), as a result of its stability in diverse light conditions. They have also assimilated the global texture model of AAM to restrict the shape and to present an optimization aspect for identifying the parameters of shape. The application of texture-constrained shape has enabled the search method to escape from the local minima of the ASM search, which consequently generates enhanced fitting outcomes. Additionally [27] have hybridized active shape models (ASM) with AAMs, in which ASMs attempt to identify appropriate landmark points using the local profile model. They have derived a gradient-based iterative method, by modifying the objective function of the ASM search, since the original objective function of the ASM search is not appropriate for combining these methods. Consequently they have proposed a new fitting method, which combines the objective functions of both ASM and AAM into a single objective function in a gradient-based optimization framework. The AAM uses gradient-based optimization techniques for model fitting, and therefore it is very sensitive to parameters of the initial model. Nevertheless, for addressing these issues, [28] have hybridized the active appearance models and the cylinder head models (CHMs), where the global head motion parameters obtained from the CHMs have been used as the cues of the AAM parameters for a good fitting or reinitialization.

The third approach for enhancing the matching performance of AAM is using a specific AAM that is suitable for different people and external variations. In this context, [29] used the person-specific AAM for tracking the subject; and they have used the generic AAM to compute subject-independent features. As the conventional AAM is generally viewpoint-specific the resulting fitting accuracy will be lower; for addressing this issue a viewpoint-specific AMM has been proposed by [30] for robust facial point extraction under multi-viewpoint. Their method collects the training samples and divides them into different groups by the viewpoint. Then, it constructs the AMMs for each training group; when a new test image is given as input, it matches the input image by all the trained AAMs, and the fitting error is computed; furthermore the minimal one will be selected as the final fitting results. When the input images have differences in pose, expression, and illumination, which were not included in the training set, the conventional AAM often deviated from giving precise outcomes. To overcome this limitation [20] have proposed tensor-based AAM, which applies multilinear algebra to the shape and appearance models of the conventional AAM, which enhances the effectiveness of fitting.

The fourth approach for enhancing the matching performance of AAM is to modify the fitting algorithm of AAM itself, by proposing a novel fitting algorithm or enhancing the existing fitting algorithms. In this perspective [14] have proposed a fast AAM using the canonical correlation analysis (CCA), which has modeled the relation between differences of the image and the model parameter for improving the convergence speed of fitting algorithm. Moreover, [31] have proposed an efficient AAM fitting algorithm based on the inverse compositional image alignment algorithm. This approach did not demand a linear relationship between the differences of image and the model parameter. However, this model has outperformed the conventional AAM in terms of yielding better fitting accuracy; furthermore the proposed model has also got faster convergence. Moreover [32] have proposed an enhanced version of fitting 2D AAM method, based on the inverse compositional image alignment algorithm, which has been employed for fitting 3D AAMs on short axis cardiac MRI. Additionally [33] have proposed 2D + 3D AAM that has an additional 3D shape model. They have also proposed the efficient fitting algorithm of 2D + 3D AAM by adding 3D constraints to the cost function of 2D AAM. On the other hand, [34] have proposed another extension of 2D + 3D AAM fitting algorithm, called multiview AAM fitting (MVAAM) algorithm, which fits a single 2D + 3D AAM to multiple view images, simultaneously obtained from multiple affine cameras. However, the evaluation process has not been adequate to demonstrate the effectiveness of the proposed approach. Similarly, [35] have proposed a new fitting algorithm of 2D + 3D AAMs for a multiple-calibrated camera system, called stereo AAM (STAAM), for increasing the fitting stability of 2D + 3D AAMs. At present, metaheuristic optimization algorithms like genetic algorithm (GA), particle swarm optimization (PSO), ant colony optimization (ACO), and artificial bee colony (ABC) have become a common solution to many optimization problems [36–38].

In this paper, we have used artificial bee colony (ABC) [39] for solving the fitting problem in AAM. The ABC algorithm simulates the intelligent foraging behavior of a honey bee swarm [40]. Nevertheless, we have not directly used the conventional ABC algorithm in our research, due to its limitation in its neighborhood search for generating new food sources. However, this issue has been addressed by introducing an adaptive ABC algorithm to solve the fitting problem in AAM. The rest of this paper is organized as follows. Firstly, the problem statement of AAM is highlighted in Section 2. Next, we have discussed the conventional and proposed method, namely, adaptive artificial bee colony in Section 3. The proposed face recognition technique is explained in Section 4. In Section 5 we have presented the results of evaluation of our proposed method. Finally, we have summarized our work in the last section.

## 2. The Problem Statement

The face recognition methods mostly use the AAM for feature extraction and recognition process, which has weaknesses in the optimization process [20]. Therefore, it is essential to enhance the optimization process in AAM by including optimization algorithm. Of late, a lot of optimization algorithms have been utilized in many research fields, but it is crucial to pick the right algorithm that suits this present study; consequently we have utilized ABC algorithm [40]. As discussed earlier, we have not directly used the conventional ABC algorithm in our face recognition technique, as it has a drawback in its neighborhood search for generating new food sources. In neighborhood search, the standard ABC algorithm randomly chooses the food position to generate the new food sources. This random generation process influences the accuracy; and this low accuracy affects the performance of recognition. Consequently, for addressing this problem we have proposed an adaptive ABC algorithm for enhancing the conventional ABC. The earlier studies related to the active appearance model have claimed that fitting the model with the target image is a very challenging task; therefor, the soft computing techniques, mostly evolutionary based algorithms, have been utilized for resolving the issue. Nevertheless, the past studies have a lack of documenting the recent developments in this domain. According to [42] the recently developed artificial bee colony (ABC) algorithm has performed well in majority of the applications. Even though [43] have already proposed an adaptive ABC algorithm, still it fails to consider the quality of current food source when it attempts to find the new food source. Hence, in this present study we have proposed an enhanced adaptive ABC algorithm, which locates the new food sources based on the quality of previous food source. However, in face recognition the fitting has to be done rapidly to handle such a huge database. Hence, in this paper, we have proposed a face recognition technique, based on active appearance model.

## 3. Artificial Bee Colony Algorithm

Swarm intelligence is an active research field, which is motivated by the combined acumen of insect or animal swarms. In the due course a number of algorithms have been proposed, which imitate the biological behaviors of insect or animal swarms, for addressing different kinds of problems; among these algorithms the artificial bee colony algorithm [42] is the most recent one, which mimics the foraging behavior of honey bee colonies. Artificial bee colony (ABC) has been widely employed for addressing multimodal and multidimensional numerical optimization problems. Generally an artificial bee colony is composed of employed bees, onlookers, and scouts. The onlooker is the one which waits on the dance area for obtaining the information about food sources, whereas an employed bee is the one which travels to the food source; and a scout is a bee that is involved in a random search. The location of a food source signifies a probable solution to the optimization problem, and the amount of nectar in a food source symbolizes the excellence of the related solution.

Initially, a distributed population is produced at random. It is noteworthy that each food source comprises only one employed bee; therefore the number of employed bees and the number of food sources will always be equal. Afterwards, the locations (solutions) might be continuously modified till the optimum solution is achieved or stop conditions are fulfilled. Fundamentally, the employed bee has the capacity to remember its earlier best location and generates a new location in its neighborhood, in its memory. Based on the greedy criterion, the employed bee upgrades its food source. Basically, when the identified new food source is superior, then the old location of food source will be updated with the new one. Once all employed bees complete their search process, they will communicate with onlookers for sharing the information, about the route and distance to food sources and the amounts of nectar; the information will be communicated by means of a waggle dance in the dancing area. By monitoring the waggle dance, each onlooker selects a food source, based on the possibility value related to the food source, and seeks the area within its neighborhood for generating a new candidate solution. Later, the greedy criterion is again applied just as it works in the employed bees. However, if the position cannot be enhanced after a prespecified number of cycles, then the position needs to be deserted; on the other hand, the corresponding employed bee turns into a scout. The abandoned position will be replaced with a new randomly generated food source [40]. The main steps can be described as follows.

1. Initialize the bee colony *X* = {*w*_*mn*1_∣*i* = 1,2,…, *mn*}, where *mn* denotes the population size and *mn*_1_ is the *i*th bee.
2. According to the fitness function, calculate the fitness *f*_*i*_ of each employed bee *w*_*mn*_ and record the maximum nectar amount as well as the corresponding food source.
3. Each employed bee produce a new solution in the nighborhood of the solution in its memory by *w*_*mn*_^new^ = *w*_min⁡_ + *∅*(*w*_min⁡_ − *w*_max⁡_), where *∅* is a random real number in [−1,1].
4. Use the greedy criterion to update *w*_*mn*_. Compute the fitness of *w*_*mn*_^new^. If *w*_*mn*_^new^ is superior to *w*_min⁡_, *w*_min⁡_ is replaced with *w*_*mn*_^new^; otherwise *w*_min⁡_ is remain.
5. According to the fitness *f*_*i*_ of *w*_min⁡_ get the probability value *P*_*i*_ via (*P*_*i*_ = fit_*i*_/∑_*i*=1_^*n*^fit_*i*_).
6. Depending on the probability *P*_*i*_ onlookers choose food sources, search the neighborhood to generate candidate solutions, and calculate their fitness.
7. Use the greedy criteria to update the food sources.
8. Memorize the best food source and nectar amount achieved.
9. Check whether there are some abandoned solutions or not. If true, replace them with some new randomly generated solutions by *w*_*mn*_ = *w*_min⁡_ + rand(0, 1)(*w*_max⁡_ − *w*_min⁡_); min and max stand for lower and upper bounds of possible solutions, respectively.
10. Repeat steps (3)–(9), until the maximum number of iterations is reached or stop conditions are satisfied.

As discussed above, the fitness function is an essential aspect of ABC algorithm, which examines the foraging quality of the colony, that is, the precision of probable solutions. Apart from this, few control parameters have to be designated, such as the number of employ bees or onlooker bees, the time limit for desertion, and the maximum number of cycles or stop conditions, which might directly impact the pace and steadiness of convergence.

Pseudocode of the ABC algorithm is given as in Algorithm 1.

### 3.1. The Proposed Adaptive ABC Algorithm

The ABC faces some inherent problems, like slow or premature convergence, particularly in case of multimodal problems like other evolutionary algorithms; in neighborhood search the standard ABC algorithm randomly chooses the food position to generate the new food sources. Generally this random generation process yields less accuracy, which negatively impacts the recognition performance. To avoid such drawbacks of the standard method, in this study we have derived an adaptive expression, which analyzes the nature of the solution space, and generates a neighborhood position as per the nectar amount of the current position.

The adaptive ABC algorithm is initiated by generating new food sources *w*_*mn*_; *m* = 0,1,…, *N*_*b*_ − 1 and *n* = 0,1,…, *N* − 1, for every *mn* employed bees, where the *w*_*mn*_ is generated using the following:

(1) $$\begin{array}{c} w_{mn}=w^{\min}+\text{rand}\left(0,1\right)\left(w^{\max}-w^{\min}\right), \end{array}$$

where min and max stand for lower and upper bounds of possible solutions, respectively. In the adaptive ABC, the neighborhood selection is done by determining new food positions as follows:

(2) $$\begin{array}{c} w_{mn}^{\text{new}}=\{\begin{array}{cc} w_{mn}+\frac{1}{w_{mn}}; & \text{if}\;w_{mn}<w_{mn}^{\text{best}},\\ w_{mn}-\frac{1}{w_{mn}}; & \text{if}\;w_{mn}>w_{mn}^{\text{best}},\\ w_{mn}; & \text{otherwise}, \end{array} \end{array}$$

where *w*_*mn*_ are the new food positions of the employee bee. These new food positions are found out by the employee bees by neighborhood search. Onlooker bees evaluate the best positions of every employee bee and go to the positions for neighborhood search as in (2). The new food sources are computed by the nectar values of the previous food sources. Initially, the current food sources nectar values are determined and subsequently the best food source, which has high nectar value among others, is found out. The higher nectar values are compared with other food sources' nectar values. If the best food sources' nectar value is greater than the other food sources' nectar values, then we increase the best food sources' nectar values; otherwise we decrease the values as shown in (2). If both values are the same, the values remain unchanged. In other words, among all the food sources, the best food sources are taken; that is, the corresponding food position is given as *w*_*mn*_^best^.

**Figure 1:**
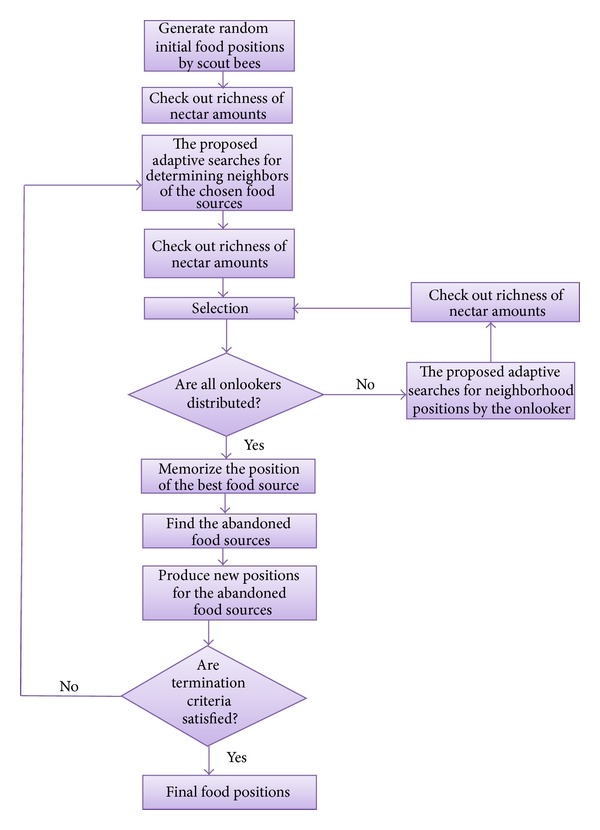
Adaptive ABC algorithm.

This means that the other food positions could be neighbors to the best food position and so the neighborhood search has to be carried in such a way that neighbor positions have to be considered. In order to reach the way, in the proposed Adaptive ABC, the current food position is either increased or decreased or kept unchanged by a factor based on the deviation between the current food position and best food position. If the current food position value is less than the best food position, the natural decision is to increase the current food position so as to reach the best food position and so the first criterion of (2) works. If the current food position value is greater than the best food position, then the current food position has to be decreased to reach the best food position and so the second criterion of (2) works. There need not be any change if the current position and the best position remain unchanged. The increment/decrement factor is decided by the current position, that is, the reciprocal of the current position. By utilizing the reciprocal, the skipping of optimal solutions can be avoided; that is, if the current position is at extreme less/high value, then the possibility of finding food positions could be more in the current position's neighbor and so by taking reciprocal a small position change will be enabled instead of going for higher position change. The worst positions are forgotten and the scout bees are sent to new random position as generated in (3). The process is iteratively repeated during a maximum swarm duration; that is, cycles are reached as shown in Figure 1.

In addition, the pseudocode of the proposed adaptive ABC approach is given in Algorithm 2.

## 4. The Proposed Face Recognition Technique

The proposed recognition technique utilizes AAM for extracting the shape and appearance features from the database images. Nevertheless, it is crucial to have appropriate fitting for extracting AAM based features. Consequently, the fitting performance has been enhanced by presenting a new adaptive ABC algorithm, where the searching performance has been accelerated by considering the quality of the best food source. Depending on the quality of the best food source, the new food sources are generated by the neighborhood search of the algorithm. The proposed technique is mainly comprised of (1) feature extraction using AAM modeling, (2) fitting using adaptive ABC, and (3) recognition phase. The three phases of the technique are discussed in Sections 4.1, 4.2, and 4.3, respectively.

### 4.1. Feature Extraction Using AAM Modeling

Given a set of training images, *I*_*i*_(*x*, *y*), *x* = 0,1,…, *n* − 1, *y* = 0,1,…, *m* − 1, and *i* = 0,1,…, *N* − 1, where *I*_*i*_(*x*, *y*) is of size *N* × *M*. In the training images, the active portions have been manually labelled for extracting the parameters of the shape and appearance models.

In a 2D image landmark points can be represented as a 2*n* shape vector, *X*, where *X* = (*x*_1_,…,*x*_*n*_, *y*_1_,…,*y*_*n*_)^*T*^. The set of shape vectors have been normalized to a common reference frame; hence *X* can be represented by *x* and by applying PCA:

(3) $$\begin{array}{c} S=\overset{-}{S}+F_{s}n_{s}, \end{array}$$

where *S* represents the synthesized shape in the normalized frame, $\overset{-}{S}$ illustrates the mean shape in the normalized frame, *F*_*s*_ depicts the matrix of eigenvectors, extracted from the training shape, and *n*_*s*_ is a set of shape parameters. Soon after acquiring a shape model, every single training image has been distorted, where its control factors could match the mean shape. Next, the texture information is tested from the shape-normalized image, which is encompassed by the mean shape for forming a texture vector, *G*. A texture model might be constructed by applying PCA to the normalized texture vectors,

(4) $$\begin{array}{c} G=\overset{-}{G}+F_{g}n_{g}, \end{array}$$

where *G* is the synthesized texture in the normalized frame, $\overset{-}{G}$ is the mean texture in the normalized frame, *F*_*g*_ is the matrix which contains eigenvectors as columns, and *n*_*g*_ is a set of texture parameters. The example of shape and texture can be synthesized by *n*_*s*_ and *n*_*g*_. Since there are correlations between shape and texture variations, a weight matrix, *W*_*s*_, should be established for each shape parameter. *W*_*s*_ is a diagonal scaling matrix. Then a concatenated vector can be generated:

(5) l=(Wsnsng)=(WsFsT(S−S−)FgT(G−G−)).

An appearance model will be set up by using a further PCA,

(6) l=Qa, Q=(QsQg),

where *Q* is the matrix which contains the eigenvectors as columns and *a* is a set of appearance parameter. Now a shape and texture model can be expressed by the appearance parameter, *a*,

(7) $$\begin{array}{c} S=\overset{-}{S}+F_{s}{W_{s}Q}_{s}a,\;\;G=\overset{-}{G}+F_{g}Q_{g}a. \end{array}$$

### 4.2. Fitting Using Adaptive ABC Algorithm

The fitting process in AAM modeling can be optimized by several existing optimization algorithms. However, due to its efficiency, ABC algorithm has been extensively used in a number of applications [44, 45] for resolving difficult optimization problems [46]. In the traditional ABC algorithm [42], novel food positions have been identified by a predefined static expression. The static expression becomes inefficient in terms of searching in huge search space, particularly in face recognition systems, because it degrades the performance of the algorithm. Consequently for enhancing the efficiency of the technique, an adaptive expression has been derived. The adaptive expression analyzes the nature of the solution space and generates a neighborhood position according to the amount of nectar in the current position (as described in the example below). The parameters that are used in the adaptive ABC algorithm are mentioned in Table 1.

The steps involved in adaptive ABC algorithm for fitting optimization are described as follows.

The adaptive ABC algorithm is initiated by generating new food sources *w*_*mn*_; *m* = 0,1,…, *N*_*b*_ − 1 and *n* = 0,1,…, *N* − 1, for every *mn* employed bees, where the *w*_*mn*_ is generated using the following:

(8) $$\begin{array}{c} w_{mn}=w^{\min}+\text{rand}\left(0,1\right)\left(w^{\max}-w^{\min}\right), \end{array}$$

where min and max stand for lower and upper bounds of possible solutions, respectively. For every food source, the nectar amount is generated by doing the steps as follows.

Firstly, generate an image model as

(9) $$\begin{array}{c} I_{m}^{\text{model}}=\left[S_{m}^{\text{model}}G_{m}^{\text{model}}\right], \end{array}$$

where

(10) $$\begin{array}{c} S=\overset{-}{S}+\xi_{i}W_{im}Q_{s}a,\\ G=\overset{-}{G}+\xi_{i}Q_{g}a, \end{array}$$

where *ξ*_*i*_ represents the eigenvalues of the image *i* and *a* is a vector of appearance parameters. Next, determine the nectar as

(11) Nm(i)=|Immodel−Iioriginal|2,

where *N*_*m*_^(*i*)^ is the nectar amount of the *i*th image. An optimal *w*_*mn*_ which can produce minimal nectar which is selected as *w*_*mn*_^best^, where *w*_*mn*_^best^ is the best food source among all the food sources and the neighborhood selection, is done by determining new food positions as follows:

(12) wmnnew={wmn+1wmn;if  wmn<wmnbest,wmn−1wmn;if  wmn>wmnbest,wmn;otherwise,

where *w*_*mn*_^new^ are the new food positions of the employee bee. These new food positions are found out by the employee bees by neighborhood search. Onlooker bees evaluate the best positions of every employee bee and go to the positions for neighborhood search as done in (12). The worst positions are forgotten and the scout bees are sent to new random position as generated in (12). The process is iteratively repeated during a maximum swarm duration; that is, cycles are reached. Once the termination criterion is met, the best weights for every image are stored in the database. The process should be explained briefly by the following example.

*Example*. We have randomly generated four food sources, namely, *x*1, *x*2, *x*3, and *x*4 with three food positions. The generated food sources are

(13) X1=11.31.4,X2=1.51.21.8,X3=1.61.91.4,X4=1.111.7.

Here, we calculate the nectar values for the generated food sources using De Jong's type I function [29] which is given in Table 2. De Jong's type I general definition is described as

(14) $$\begin{array}{c} f\left(x\right)=\overset{n}{\underset{i=1}{\sum }}{x_{i}}^{2}, \end{array}$$

where in (14) *x*_*i*_ range is −5.12 ≤ *x*_*i*_ ≤ 5.12, *i* = 1,…, *n*.

*Conventional ABC Algorithm*. The new solutions are generated for the above food sources by the conventional ABC algorithm neighborhood search. The neighborhood search formula is stated as follows:

(15) $$\begin{array}{c} w_{mn}^{\text{new}}=w_{\min}+\emptyset \left(w_{\min}-w_{\max}\right). \end{array}$$

The generated new food sources are

(16) X1=0.921.461.24X2=1.91.041.8,X3=1.762.221.32,X4=1.020.442.26.

The computed nectar values are given in Table 2. The nectar values of food sources of the conventional ABC algorithm are greater than the nectar values of initially generated food sources.

*Adaptive ABC Algorithm*. For the given initial food sources, the new solutions are generated by our proposed adaptive ABC algorithm. The new solutions are generated by using (12) and the new solutions are

(17) X1=11.31.4,X2=0.832.036.38,X3=0.981.371.40,X4=0.1921.11.

**Figure 2:**
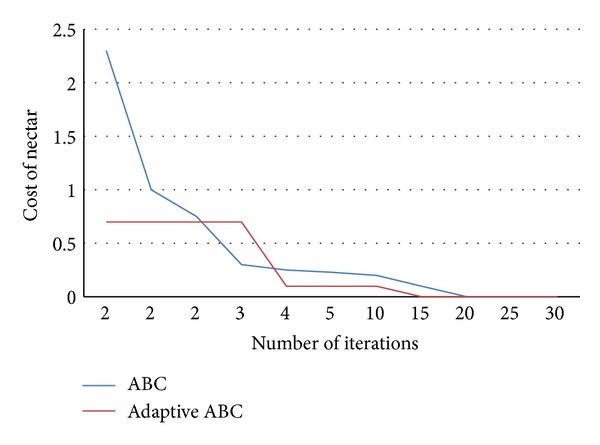
Adaptive and conventional ABC algorithms performance in terms of their nectar values.

In Table 2, *N*(*x*1) is the nectar value of the food source *X*_1_. As can be seen from Table 2, our proposed adaptive ABC algorithm has generated new food sources with minimum nectar values. Based on the above example the performances of conventional and proposed adaptive ABC algorithms are given in Figure 2.

Figure 2 presents the nectar cost representation when conventional and adaptive ABCs are implemented. One can see from the graph that, in conventional ABC, the bees take time to find rich food positions with higher cost as compared to the bees of proposed ABC.

### 4.3. Recognition Phase

The recognition system acknowledges the credibility of an image by evaluating the parameters of the image with the images in the database. The presence of image in the database assures the credibility, and the absence of images forbids the credibility. Furthermore, the test image is exposed to the extraction of shape and appearance parameter, in order to perform the recognition. The recognition system performs similarity measure using distance measure formula as given in (18) and makes decision:

(18) Ntest=∑x=0M−1∑y=0N−1I(x,y)original−I(x,y)model.

*N*_test_ is obtained for the test image that is compared between the test and database image. The decision on authenticity is taken as follows:

(19) Person  ID={Anonymous;if  Ntest>NTH,I;otherwise,

where

(20) $$\begin{array}{c} I=\arg_{i}\min\left(N_{{\text{test}}_{i}}\right). \end{array}$$

The decision making system outputs the person ID if the subjected test unknown image has authenticity and Unidentified if has not the authenticity.

## 5. Experimental Results

**Figure 3:**
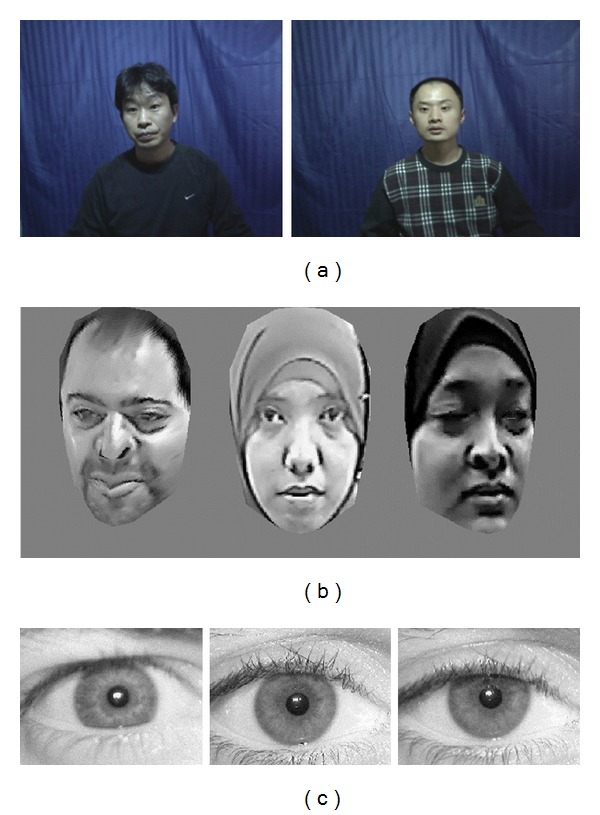
Sample images from (a) CASIA face dataset, (b) 2.5D face dataset, and (c) UBIRIS iris dataset.

The performance of our proposed method has been analyzed in three evaluation steps: (1) it evaluated the performance in terms of recognition results; (2) it evaluated the fitting solution adaptive ABC against ABC and (3) evaluated the proposed AAM method using fitting errors. The proposed recognition system has been experimented in the working platform of MATLAB 7.12 with the system configuration, i5 CPU @ 3.19 GHz with 4 GB RAM; and evaluation has been done using CASIA-Face V5 database [47] and our own 2.5D face dataset collected by our cybersecurity group. Moreover, UBIRIS dataset [48] has also been used to validate the stability of the proposed method with other types of biometric categories.  Figure 3 shows some examples from the three datasets: CASIA, 2.5D and UBiris respectively. From CASIA database, 500 images have been used and then have been divided into five parts for experimentation. In each part, there are 100 images at five different environments of poses and illumination variations. For our property 2.5D dataset and UBIRIS dataset, 121 images are utilized in training and 6 images are exploited in testing. The performance of the technique is analyzed by conducting* n*-fold (for all datasets, *n* = 10) cross validation over all datasets and the corresponding statistical performance measures are determined. To perform* n*-fold cross validation, tenfolds of training and testing datasets are generated by folding operation.

### 5.1. Performance Evaluation Using Recognition Results

**Figure 4:**
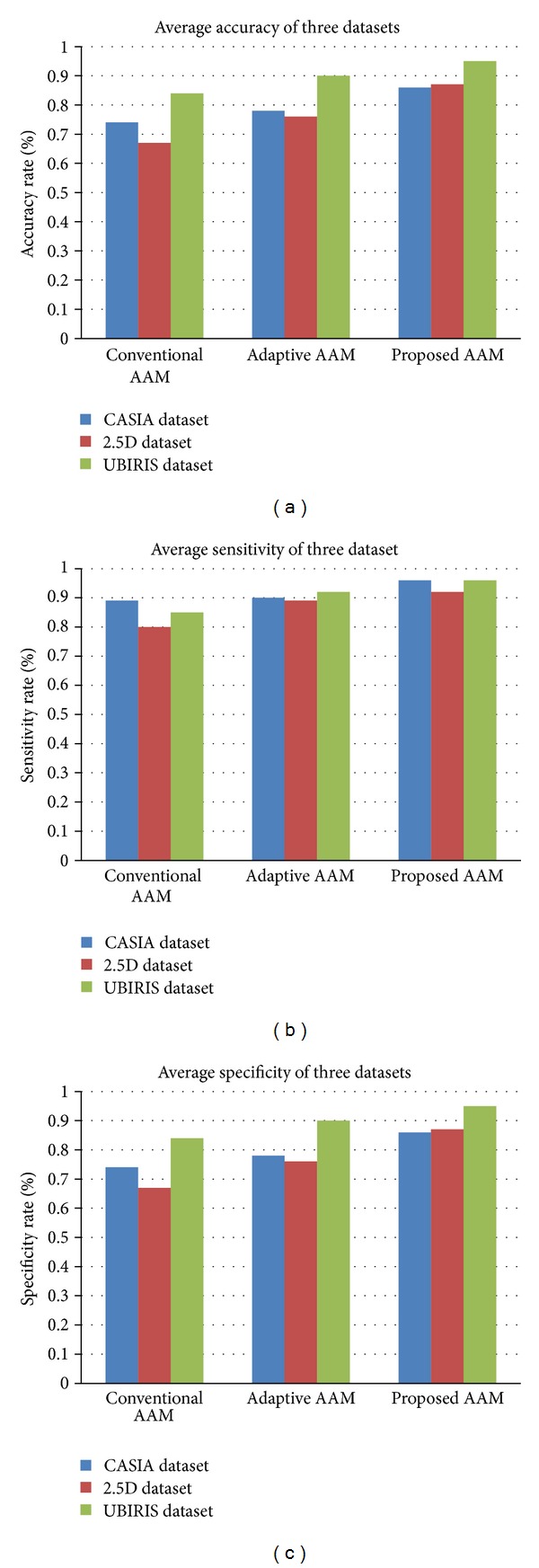
Average of ten cross validation results recognition performance for three datasets in terms of (a) accuracy, (b) sensitivity, and (c) specificity for conventional AAM, adaptive AAM, and the proposed AAM.

The performance of the proposed technique has been analyzed by conducting* n*-fold (for our dataset, *n* = 10) cross validation over all datasets and the corresponding statistical performance measures are determined. The comparison is done with the conventional AAM and with the adaptive AAM [41]. The cross validation results for 1 :* N* recognition over three datasets are tabulated in Tables 3, 4, 5, 6, 7, 8, 9, 10, and 11. The statistical and the average recognition performance of the three datasets is illustrated in Figure 4. Performance that measures accuracy, sensitivity, and specificity is defined as follows.

 Accuracy: accuracy represents the degree of closeness of measurements of a quantity with its authentic (true) value.
 Sensitivity: sensitivity measure gives the percentage of recognized face images that are correctly identified as recognized face image.
 Specificity: specificity measure gives the percentage of nonrecognized face images that are correctly identified as nonrecognized face image.

### 5.2. Fitting Optimization: ABC versus Adaptive ABC

**Figure 5:**
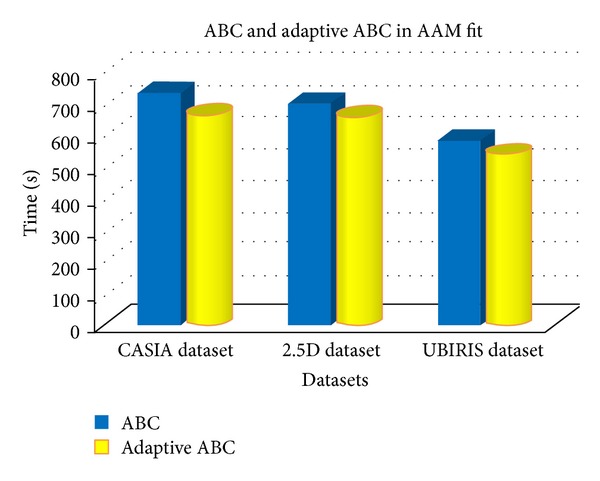
The average time taken to fit the model in the original image by conventional and adaptive ABC over the three datasets.

**Figure 6:**
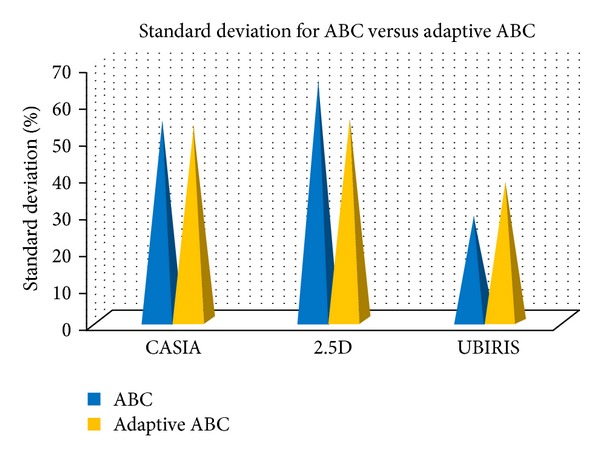
Standard deviation taken by conventional and adaptive ABC algorithm for the three datasets.

In order to evaluate the efficiency of the proposed adaptive ABC, the three datasets CASIA, 2.5D, and UBIRIS, respectively, have been tested and evaluated in 10 rounds. The proposed adaptive ABC outperformed the conventional ABC in optimization process in order to create a proper fitting of model over the original image. The performance of adaptive ABC as against the conventional ABC over the three datasets is tabulated and illustrated in Table 12 and Figures 5 and 6, respectively.

Moreover, the best food sources obtained from different iterations are illustrated in Figure 8.

**Figure 7:**
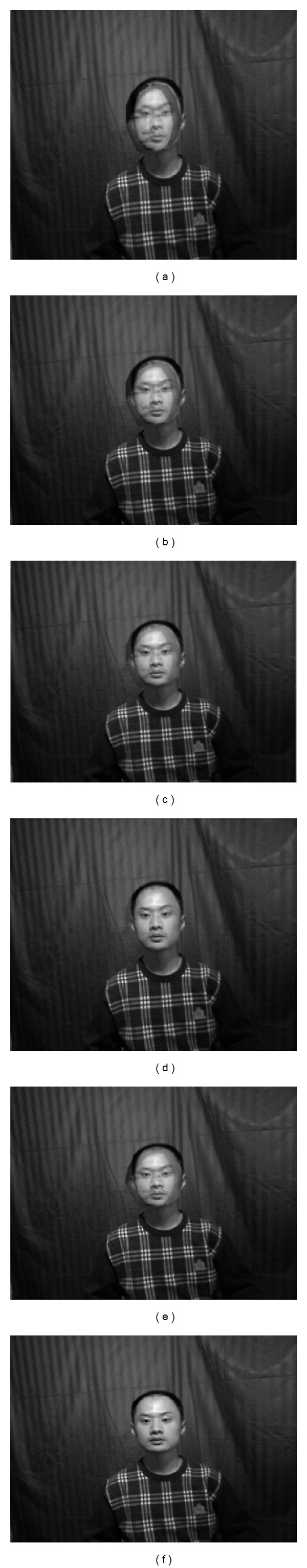
Fitting model example of faces at (a) iteration 1, (b) iteration 2, and (c) iteration 3, (d) final fit model, (e) standard AAM image, and (f) original image.

**Figure 8:**
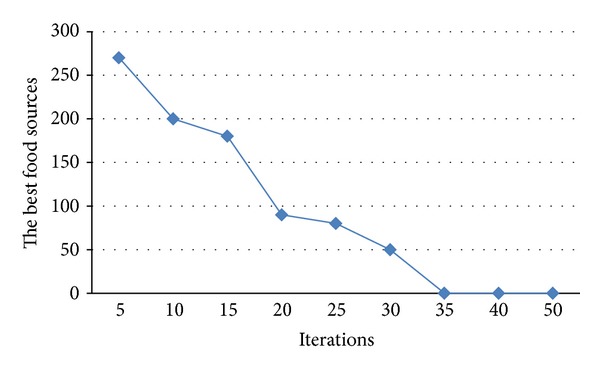
Graphical representation of the best food sources with number of iterations.

*Discussion*.  Figure 7 depicts the image outputs of fitting performance under different iterations. When the result of ABC algorithm is compared with the results of adaptive ABC algorithm, based on different three datasets with different random ten runs, the *t*-test result shows that the proposed method is considered to be statistically significant and has outperformed the standard method with the *t*-test result (*P* < 0.05 and *P* = 0.008, 0.0024, and 0.0016, resp., for the three datasets). The adaptive ABC algorithm relatively takes less time than the conventional model of ABC algorithm, except for the third cross validation round of third dataset. In order to make a conclusion over the performance, the mean value has been taken for all the rounds and plotted in figure. Based on the results it is evident that the proposed adaptive ABC has outperformed the standard ABC in terms of achieving fitting efficiency, as the deficiency of performance in the third round of the third dataset seems to be negligible when compared to the performance over the other rounds of the datasets. When comparatively analyzing the datasets, the mean value has clearly showed that the adaptive ABC has consumed less time than conventional ABC algorithm, in terms of fitting the model. Furthermore, the standard deviation has showed similar result, except for the UBIRIS dataset. Generally, adaptive ABC is more efficient than the conventional ABC while dealing with the AAM fitting problem.

### 5.3. Evaluating the Proposed AAM Method Using Fitting Errors

The mean square error (MSE) is the average of the squared errors between target image and estimated model readings in a data sample:

(21) $$\begin{array}{c} \text{Mse}=\frac{1}{n}\overset{n}{\underset{i=1}{\sum }}{\left(\text{model}-\text{image}\;\right)}^{2}. \end{array}$$

**Figure 9:**
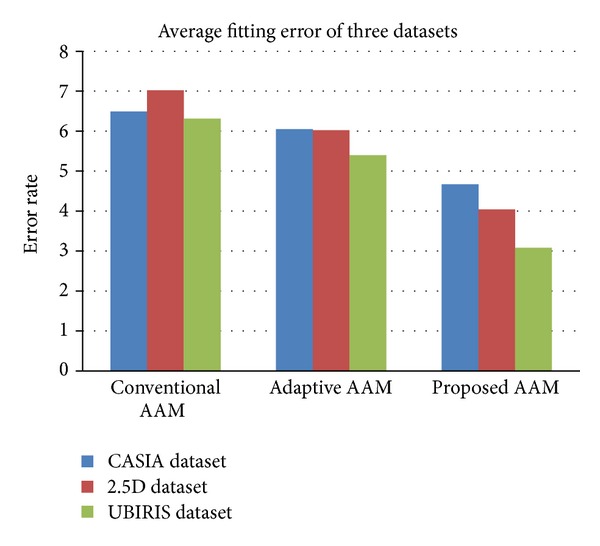
Fitting error average for 10 rounds taken from fitting the model in the original image by conventional AAM, adaptive AAM, and the proposed AAM.

For example, if 100 landmarks have fitted over the target image in one of the rounds and 95 landmark points are exactly fit in the same target positions. This mean 5 landmarks might represent error 100 − 95 = 5 because fitting will not be converged until it goes to zero. Five points may not reach the target coordinates. If 5 points out of 100 points fail to exactly reach the target coordinates, so the fitting error would be 5/100 = 5%. To analyze and evaluate the performance using MSE, the performance of 10 rounds of experiments has been conducted over the three datasets. The results of fitting error values of 10 rounds of experiments for conventional AAM, adaptive AAM, and the proposed AAM techniques have been presented, respectively, in Table 13 and Figure 9. In addition, Table 14 shows the *t*-test results for the proposed AAM as against the conventional AAM and adaptive AAM, in terms of accuracy and fitting error from the three datasets.

## 6. Conclusion

In this paper, a face recognition technique had been proposed by extracting AAM based features. The AAM fitting problem had been solved by introducing a new adaptive ABC algorithm, in which the neighborhood selection has been accelerated by considering the nature of current food position. The introduction of adaptive ABC has fastened the AAM fitting, and hence the efficiency of recognition has been improved without compromising the recognition performance. The performance of the technique had been analyzed by using three datasets, such as CASIA face database version 5, property 2.5D face dataset, and UBIRIS dataset. The efficiency of recognition had been evaluated by experimenting 1 :* N* face recognition problem. The experimental results had proved that the proposed technique has been statistically significant in terms of aforesaid features. In addition, the fitting error between the generated model and target image shows that the proposed AAM had been more efficient than the conventional AAM approaches. Moreover, the graphical illustration of fitting efficiency of ABC over adaptive ABC had revealed the improvement in terms of recognition efficiency. Eventually, we have concluded that the adaptive ABC improves the recognition efficiency without compromising the accuracy.

## Figures and Tables

**Algorithm 1:**
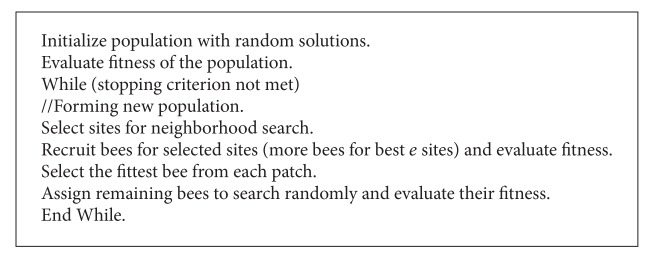


**Algorithm 2:**
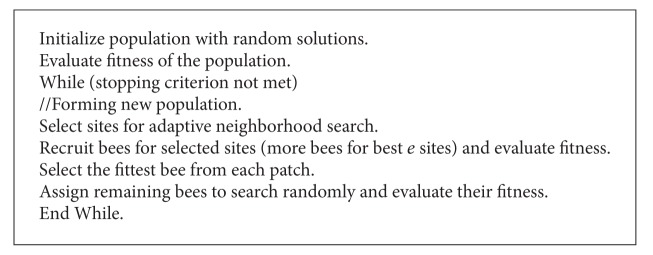


**Table 1:** Parametric values used for adaptive ABC.

Parameters	Values
Population size	10
Number of scout bees	10
Number of *n* sites	10
Number of selected sites out of *n* sites	5
Number of *m* sites	3
Number of the best sites out of *m* selected sites	1

**Table 2:** Computed nectar values.

Nectar values from De Jong's type I	Nectar values from conventional ABC algorithm	Nectar values from adaptive ABC algorithm
*N*(*x*1) = 4.65	*N*(*x*1) = 4.51	*N*(*x*1) = 4.65
*N*(*x*2) = 6.93	*N*(*x*2) = 7.93	*N*(*x*2) = 6.38
*N*(*x*3) = 8.13	*N*(*x*3) = 9.77	*N*(*x*3) = 4.80
*N*(*x*4) = 5.1	*N*(*x*4) = 6.34	*N*(*x*4) = 5.27

**Table 3:** Cross validation results for CASIA face dataset in terms of accuracy for conventional AAM, adaptive AAM, and the proposed AAM.

Accuracy of CASIA face dataset			
Cross validation rounds	Conventional AAM	Adaptive AAM [41]	Proposed AAM
1	0.7	0.9	0.8
2	0.8	0.8	0.9
3	0.9	0.9	1
4	0.7	0.8	0.9
5	0.6	0.7	0.8
6	0.8	0.9	1
7	0.6	0.7	0.9
8	0.9	0.8	0.9
9	0.8	0.7	0.8
10	0.7	0.7	0.8

Average	0.75	0.79	0.88

**Table 4:** Cross validation results for 2.5D face dataset in terms of accuracy for conventional AAM, adaptive AAM, and the proposed AAM.

Accuracy of property 2.5D face dataset			
Cross validation rounds	Conventional AAM	Adaptive AAM [41]	Proposed AAM
1	0.7	0.9	0.9
2	0.8	0.8	0.9
3	0.7	0.8	0.8
4	0.8	1	0.9
5	0.7	0.7	0.8
6	0.78	0.8	1
7	0.8	0.85	0.9
8	0.75	0.8	0.8
9	0.85	0.8	0.95
10	0.9	0.9	1

Average	0.78	0.84	0.9

**Table 5:** Cross validation results for UBIRIS dataset in terms of accuracy for conventional AAM, adaptive AAM, and the proposed AAM.

Accuracy of UBIRIS dataset			
Cross validation rounds	Conventional AAM	Adaptive AAM [41]	Proposed AAM
1	0.88	0.95	1
2	0.9	0.95	0.9
3	0.8	0.9	0.95
4	0.9	0.85	0.9
5	0.8	0.9	0.9
6	0.7	0.75	0.7
7	0.9	0.9	1
8	0.85	0.85	0.9
9	0.75	0.9	0.8
10	0.95	1	1

Average	0.84	0.90	0.91

**Table 6:** Cross validation results for CASIA face dataset in terms of sensitivity for conventional AAM, adaptive AAM, and the proposed AAM.

Sensitivity of CASIA face dataset			
Cross validation rounds	Conventional AAM	Adaptive AAM [41]	Proposed AAM
1	0.8	0.9	0.8
2	1	1	1
3	0.9	0.9	1
4	1	0.7	0.8
5	0.9	0.9	1
6	0.8	1	1
7	0.8	0.9	1
8	0.9	0.9	1
9	0.8	1	1
10	1	0.8	1

Average	0.89	0.9	0.96

**Table 7:** Cross validation results for 2.5D face dataset in terms of sensitivity for conventional AAM, adaptive AAM, and the proposed AAM.

Sensitivity of 2.5D face dataset			
Cross validation rounds	Conventional AAM	Adaptive AAM [41]	Proposed AAM
1	0.75	0.8	0.85
2	0.8	0.95	1
3	0.8	0.85	0.7
4	0.9	1	1
5	0.7	0.9	0.8
6	1	1	0.9
7	0.7	0.8	1
8	0.6	0.9	0.9
9	0.8	0.9	1
10	0.95	0.8	1

Average	0.8	0.89	0.92

**Table 8:** Cross validation results for UBIRIS dataset in terms of sensitivity for conventional AAM, adaptive AAM, and the proposed AAM.

Sensitivity of UBIRIS dataset			
Cross validation rounds	Conventional AAM	Adaptive AAM [41]	Proposed AAM
1	0.9	1	1
2	0.7	1	1
3	1	0.8	0.9
4	0.95	0.9	0.9
5	0.8	0.9	1
6	1	1	0.95
7	0.85	0.95	1
8	0.8	0.95	0.9
9	0.7	0.8	1
10	0.8	0.9	0.9

Average	0.85	0.92	0.96

**Table 9:** Cross validation results for CASIA face dataset in terms of specificity for conventional AAM, adaptive AAM, and the proposed AAM.

Specificity of CASIA face dataset			
Cross validation rounds	Conventional AAM	Adaptive AAM [41]	Proposed AAM
1	0.8	0.7	0.8
2	0.6	0.7	0.8
3	0.8	0.9	1
4	0.8	0.7	1
5	0.7	0.7	0.6
6	0.7	0.8	1
7	0.6	1	0.8
8	0.8	0.8	1
9	0.9	0.9	1
10	0.7	0.6	0.6

Average	0.74	0.78	0.86

**Table 10:** Cross validation results for 2.5D dataset in terms of specificity for conventional AAM, adaptive AAM, and the proposed AAM.

Specificity of 2.5D face dataset			
Cross validation rounds	Conventional AAM	Adaptive AAM [41]	Proposed AAM
1	0.8	0.9	0.95
2	0.9	0.9	0.9
3	0.7	0.7	0.9
4	0.8	0.7	1
5	0.8	0.7	0.7
6	0.7	0.8	0.9
7	0.9	0.8	0.95
8	0.7	0.6	0.7
9	0.6	0.7	0.8
10	0.8	0.8	0.9

Average	0.67	0.76	0.87

**Table 11:** Cross validation results for UBIRIS in terms of specificity for conventional AAM, adaptive AAM, and the proposed AAM.

Specificity of UBIRIS dataset			
Cross validation rounds	Conventional AAM	Adaptive AAM [41]	Proposed AAM
1	0.9	0.95	1
2	0.8	1	1
3	0.8	0.9	1
4	0.9	0.95	1
5	0.9	0.8	0.9
6	0.8	0.9	0.9
7	0.9	0.9	0.9
8	0.7	0.7	0.8
9	0.8	0.9	1
10	0.9	0.95	0.95

Average	0.84	0.9	0.95

**Table 12:** Mean time (in seconds) and its standard deviation for the three datasets taken by conventional and adaptive ABC algorithm for the best fitting process.

Rounds	ABC algorithm			Adaptive ABC algorithm		
	CASIA dataset	2.5D dataset	UBIRIS dataset	CASIA dataset	2.5D dataset	UBIRIS dataset
1	674.8	589.7	570.8	622	540.3	502.4
2	693.8	620.9	530.6	618.6	618	518.8
3	742.4	698.2	612.2	668.8	640.9	614
4	791.6	790	602.5	700.2	702.2	570.7
5	786.8	730.7	598	707	692.5	534.6
6	726.6	680.4	615.9	661.6	633.6	566
7	674.8	665.8	584.7	611.4	680	577.2
8	697.2	733.9	596.4	643.4	714.7	512.9
9	753.6	720.5	607	640.6	677.8	518.6
10	836.2	790.7	543.9	786.2	720.4	501.3

Mean	737.78	702.08	586.2	665.98	662.04	541.65

STD	54.667	65.638	29.097	53.38	55.08	38.05

**(a):** 

Fitting error			
Experiments	Conventional AAM	Adaptive AAM [41]	Proposed AAM
1	6	6.82	4
2	7.4	8.57	6.42
3	6	5.62	5.22
4	7.2	5.75	5.29
5	8	4	6.40
6	6.5	4.87	4
7	4	6	3.12
8	5	7	2.93
9	7.8	5.84	4.33
10	7	6	5

Average	6.49	6.05	4.67

**(b):** 

Fitting error			
Experiments	Conventional AAM	Adaptive AAM [41]	Proposed AAM
1	7	5.93	3
2	6.8	7.43	4
3	8	6.93	5
4	8.8	4.45	5.8
5	7	5	6
6	5.7	5.54	4
7	8	7	2.58
8	6	5	3
9	5.9	6.92	4
10	7	6	3

Average	7.02	6.02	4.038

**(c):** 

Fitting error			
Experiments	Conventional AAM	Adaptive AAM [41]	Proposed AAM
1	6	5	2
2	5	6	3
3	6	7	1
4	8	3	4.87
5	8	4	4
6	6	8	3
7	5	5	5
8	4.43	4	2
9	6.87	6	2.95
10	7.84	6	3

Average	6.314	5.4	3.082

**(a):** 

*t*-test results on the three datasets in terms of accuracy		
Dataset	Proposed AAM versus conventional AAM	Proposed AAM versus adaptive AAM [41]
CASIA	0.0019	0.0039
2.5D	0.017	0.004
UBIRIS	0.0053	0.619

**(b):** 

*t*-test results on the three datasets in terms of fitting error		
Dataset	Proposed AAM versus conventional AAM	Proposed AAM versus adaptive AAM [41]
CASIA	0.0042	0.0218
2.5D	1.1864	0.0082
UBIRIS	1.3105	0.0132
